# Mechanisms of Impact of Blue Spaces on Human Health: A Systematic Literature Review and Meta-Analysis

**DOI:** 10.3390/ijerph18052486

**Published:** 2021-03-03

**Authors:** Michail Georgiou, Gordon Morison, Niamh Smith, Zoë Tieges, Sebastien Chastin

**Affiliations:** 1 School of Health and Life Sciences, Glasgow Caledonian University, 70 Cowcaddens Road, Glasgow G4 0BA, UK; Niamh.Smith@gcu.ac.uk (N.S.); zoe.tieges@gcu.ac.uk (Z.T.); sebastien.chastin@gcu.ac.uk (S.C.); 2 School of Engineering and Built Environment, Glasgow Caledonian University, 70 Cowcaddens Road, Glasgow G4 0BA, UK; Gordon.Morison@gcu.ac.uk; 3 Geriatric Medicine, Usher Institute, University of Edinburgh, 51 Little France Crescent, Edinburgh EH16 4SA, UK; 4 Department of Movement and Sports, Ghent University, Watersportlaan 2, 9000 Ghent, Belgium

**Keywords:** physical activity, stress, social isolation, pollution, heat island, urban nature, park, lake, health, environment

## Abstract

Blue spaces have been found to have significant salutogenic effects. However, little is known about the mechanisms and pathways that link blue spaces and health. The purpose of this systematic review and meta-analysis is to summarise the evidence and quantify the effect of blue spaces on four hypothesised mediating pathways: physical activity, restoration, social interaction and environmental factors. Following the PRISMA guidelines, a literature search was conducted using six databases (PubMed, Scopus, PsycInfo, Web of Science, Cochrane Library, EBSCOHOST/CINAHL). Fifty studies were included in our systematic review. The overall quality of the included articles, evaluated with the Qualsyst tool, was judged to be very good, as no mediating pathway had an average article quality lower than 70%. Random-effects meta-analyses were conducted for physical activity, restoration and social interaction. Living closer to blue space was associated with statistically significantly higher physical activity levels (Cohen’s d = 0.122, 95% CI: 0.065, 0.179). Shorter distance to blue space was not associated with restoration (Cohen’s d = 0.123, 95% CI: −0.037, 0.284) or social interaction (Cohen’s d = −0.214, 95% CI: −0.55, 0.122). Larger amounts of blue space within a geographical area were significantly associated with higher physical activity levels (Cohen’s d = 0.144, 95% CI: 0.024, 0.264) and higher levels of restoration (Cohen’s d = 0.339, 95% CI: 0.072, 0.606). Being in more contact with blue space was significantly associated with higher levels of restoration (Cohen’s d = 0.191, 95% CI: 0.084, 0.298). There is also evidence that blue spaces improve environmental factors, but more studies are necessary for meta-analyses to be conducted. Evidence is conflicting on the mediating effects of social interaction and further research is required on this hypothesised pathway. Blue spaces may offer part of a solution to public health concerns faced by growing global urban populations.

## 1. Introduction

The world’s urban population has grown by approximately 460% between 1950 and 2018, increasing the number of people living in urban areas from 751 million in 1950 to 4.2 billion in 2018 [1]. This tremendous increase in the urban population has raised several environmental, social and health concerns [2]. Urbanisation is linked to increased risk of non-communicable diseases, premature mortality [3], as well as a higher risk of mental illnesses [4] and social isolation [5]. Urban growth is projected to continue and bring an additional 2.5 billion people to urban areas by 2050 [1]. It is therefore of paramount importance for city-planners to create sustainable and healthy urban environments, which promote mental and physical wellbeing.

Natural environments bring several benefits to public health and social wellbeing in urban settings. Studies have shown that exposure to natural environments contributes to reduced mortality rates and increased wellbeing among urban dwellers [6]. Most of the research has concentrated on the impact of green spaces (e.g., parks), but in recent years it has emerged that blue spaces such as coasts, lakes, rivers and canals can bring similar benefits [7,8,9]. To date, few studies differentiate between green and blue spaces, as blue space is often treated as an inherent component of parks and natural environments [9]. However, blue spaces are independent entities and there is a need to be considered separately and not solely as a subcategory of green spaces [10]. Over the years, research has focused on the negative effects of blue spaces and the understanding of such effects is well developed [11]. Health hazards, such as an increased risk of flooding and higher levels of disease transmission, through exposure to several microbes and contact of humans with a wide range of hazardous chemicals, have often been linked to blue spaces [11]. However, recent epidemiological studies have shown that blue spaces also have a positive effect on public health [9], including the reduction of mortality rate with the greatest rate of decline seen in areas closest to blue space [12], better physical health [7], and better mental health [8]. In fact, a recent meta-analysis quantified the health impact of blue spaces and concluded that it is as strong as that of green spaces [9]. Therefore, it logically derives that the existence of such benefits from blue spaces also enables discussion of environmental justice around their accessibility and availability to some groups of the population. Simultaneously, blue spaces are considered valuable ecosystem services, have both an aesthetic and ecological role in urban environments and can be used for urban microclimate regulation [10,13].

**Figure 1:**
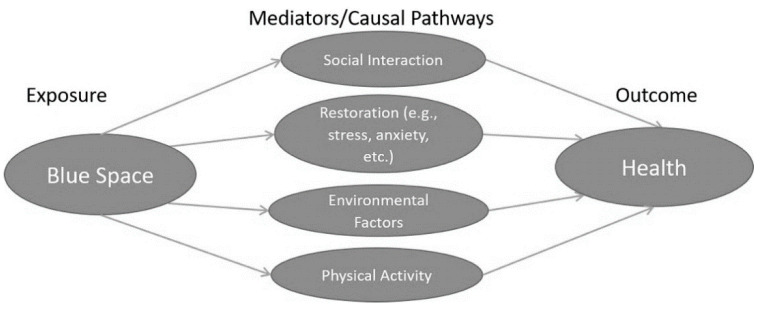
Blue Space—Hypothesised Health Mediators/Causal Pathways.

In order to leverage these salutogenic effects and improve the health of the urban population, it is important to understand the linking mechanisms between exposure to blue space and health. Four mechanisms have been proposed to mediate the relationship between blue spaces and health (Figure 1): (1) Access to blue spaces may promote physical activity which is the fourth most important risk factor for poor health [14]; (2) Exposure to blue spaces may improve restoration [15]. This follows the definition by [15] and therefore considers markers of restoration, including, but not limited to, stress, anxiety, depressed mood and psychological wellbeing, which have been linked with risk of cardiovascular diseases [16] and mental health issues [17]; (3) Blue spaces may contribute to a healthier environment and reduce air pollution, heat island effect, risk of flooding [18]; and finally (4) Blue spaces may promote social interactions which have been found to benefit mental and physical health, among others, through a sense of community, mutual support between people, quicker emergency reaction and sense of coherence [19].

To date there has been no review synthesising evidence about these potential mechanisms.

The purpose of this systematic review and meta-analysis is to summarise current evidence and quantify the effect of blue space on physical activity, restoration, environmental factors and social interaction.

## 2. Materials and Methods

This systematic review and meta-analysis followed the PRISMA guidelines and the composition of systematic reviews in research guidelines [20,21]. The review protocol was pre-registered with PROSPERO (available at http://www.crd.york.ac.uk/PROSPERO/ (accessed on 22 January 2021) with registration number CRD42019154917).

### 2.1. Search Strategy

Six databases (PubMed, Scopus, PsycInfo, Web of Science, Cochrane Library, CINAHL (EBSCO)) were searched for articles using keywords and synonyms of terms pertaining to urban green and blue spaces (e.g., rivers, canals) and potential mechanisms or mediating factors (e.g., physical activity, stress, sleep, air pollutant, social interaction, noise). For each database, a search string was created, combining these keywords (search strategy provided in Supplementary File/Table S2). Searches were limited to articles reporting research on human participants and published in English from inception until 22 January 2021. A snowball search for relevant studies was conducted, by two reviewers (MG, SC), based on the reference lists provided in the included articles of this review and review articles identified. Explanation of search terms is provided in Supplementary File/Table S1.

### 2.2. Eligibility Criteria

To be included in this review and meta-analysis the studies had to fulfil the inclusion and exclusion criteria detailed in Table S3 in the supplementary material. Briefly, studies had to present original peer-reviewed research providing quantitative information about the relationship between exposure to blue spaces and markers of social interaction, restoration, physical activity and/or environmental factors. We included studies which considered those as outcomes or mediators. The following blue spaces were considered: all inland waterways, coastal environments, canalled areas, blue infrastructure (BI), navigable transportation canals, aqueducts, lakes, marinas, rivers, ponds, reservoirs, marshes, estuaries, fountains, streams, reconstructed or recalibrated wetlands, waterfront parks, deculverted/daylighted areas, open air streams, urban waterways, riparian corridors, recalibrated urban parks, urban forests, natural preserves. Included studies had to be of the following designs: cross-sectional, longitudinal, cohort study, case study of specific sites, natural experiment, prospective study, randomised controlled trial, case reports and series, cross-over study, or evaluation study. We considered studies that reported exposure to blue space in the following categories: distance to blue space, amount of blue space within a geographical area, contact with blue spaces (e.g., visits) and visibility of blue space.

Studies were excluded if they were: qualitative studies, opinion pieces, theoretical papers, non-peer-reviewed or conducted using a virtual environment.

### 2.3. Screening, Data Extraction and Quality Appraisal

All search results/articles were retrieved and uploaded to the Rayyan QCRI online tool for systematic reviews [22]. Study abstracts and titles were independently screened for inclusion by two reviewers from a pool of four (MG, SC, ZT, NS). A third reviewer (out of the reviewer pool) was used to resolve disagreement where necessary. Full-text screens were then carried out independently by two reviewers (MG, SC), while a third reviewer was used to settle conflicting decisions.

For data extraction, a standard template was used, containing details of each article’s title, author, date, title, population, age (mean (SD)), sample size, design, main results, area/context, blue space exposure, method of blue space exposure measurement, and confounding variables.

Quality appraisal of studies was conducted by two reviewers using the Standard Quality Assessment Criteria for Evaluating Primary Research Papers from a Variety of Fields QUALSYST tool [23]. This tool was chosen as it enables the assessment of quality and evaluation of potential bias over a wide range of research designs from experimental to observational [24]. All articles were evaluated on a rating scale in five domains: use of correct methods, design appropriateness, sample size, inclusion of confounding variables, report of sufficient statistical evidence and description of participants/subjects.

### 2.4. Meta-Analyses

Studies were classed according to how exposure to blue space was measured (e.g., distance to blue space, amount of blue space, frequency of visits) and the mechanism/mediator investigated, by two study authors (MG, SC). A meta-analysis was conducted when at least three studies were available for the same exposure and mechanism/mediator combined. Meta-analyses were feasible for the association between the amount of blue space and physical activity, distance to blue space and physical activity, amount of blue space and restoration, distance to blue space and restoration, contact with blue space and restoration and distance to blue space and social interaction. Other categories did not have a sufficient number of articles or did not report sufficient statistics to permit a meta-analysis. Prior to each meta-analysis, the effect size of blue space of each study was extracted and converted to Cohen’s d, based on conversion methods for effect sizes in the existing literature [25,26]. Effect sizes were pooled using a random-effect model meta-analysis, and the results were presented as forest plots. We interpreted Cohen’s d effect sizes as low, moderate, or high, according to upper limits of 0.2, 0.5 and 0.8, respectively [27]. Subsequently, when a study used several different measurements for the same exposure category, the same outcomes reported by this study were averaged over the different measurement in the same exposure category. For studies reporting separate results for different groups for the same exposure and outcome, we computed the average outcome for each exposure category weighted by the sample size of each group. When studies reported the same outcome measure both objectively and via self-report, we prioritised the objective measure. For example, Garrett et al. [28] reported both self-reported physical activity and accelerometer physical activity levels. In this case, the objective measure (accelerometer) was prioritised over the self-reported physical activity levels. Heterogeneity amongst studies was gauged by visual inspection of funnel plots and quantified using I^2^ statistics. With upper limits of 25%, 50% and 75% respectively for I^2^, heterogeneity was interpreted as medium, moderate, or high [29]. All meta-analyses were computed using Comprehensive Meta-Analysis software version 3 [30]. Forest plots were created based on Cohen’s d effect size, using the same software [30].

## 3. Results

**Figure 2:**
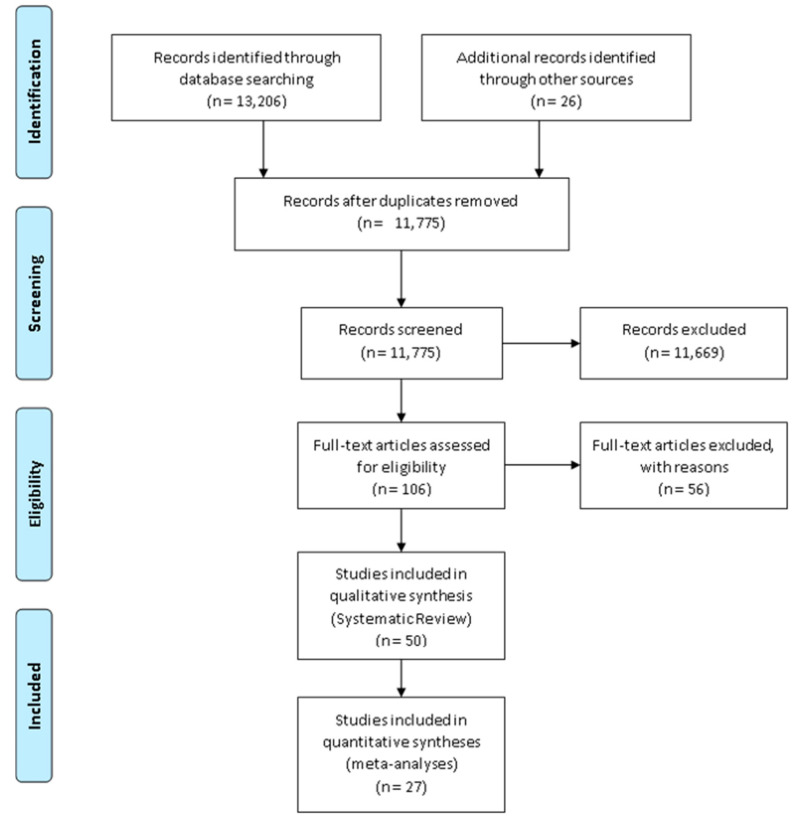
PRISMA Flow Diagram.

The electronic searches identified 13,206 articles; 9122 in PubMed, 47 in Scopus, 1136 in PsycInfo, 1843 in Web of Science, 53 in Cochrane Library, and 1005 in CINAHL (EBSCO). 26 more papers, meeting the inclusion criteria, were added to the database from the snowball search. After removing duplicates, 106 articles were found to be eligible for full-text screening. This resulted in 50 studies being included in the review. The main reasons for excluding studies were that studies did not measure the right exposure, were qualitative, were conducted in a virtual environment or referred solely to impacts of green space. The data flow is presented in Figure 2. All 50 articles were split into four categories based on their mediating pathways, while eight articles presented findings for more than one mediating pathway and these were therefore assigned to more than one category. There were 18 articles for physical activity, 21 for restoration, seven for social interaction, and 14 articles for environmental factors.

### 3.1. Study Design Characteristics

Among the 50 articles included in this review, 35 articles had a cross-sectional design [31,32,33,34,35,36,37,38,39,40,41,42,43,44,45,46,47,48,49,50,51,52,53,54,55,56,57,58,59,60,61,62,63,64,65], four were cross-over studies [66,67,68,69], seven were of longitudinal design [70,71,72,73,74,75,76], two were cohort studies [77,78], one article had both a longitudinal and cross-sectional design [79] and one article had both a cross-over and cross-sectional design [28] (Table 1). For physical activity, 14 articles were cross-sectional [28,31,32,33,34,35,36,37,38,55,56,57,58,59], one longitudinal [70], two cohort studies [77,78] and one both cross-over and cross-sectional [28]. For restoration, 14 articles were cross-sectional [39,40,41,42,43,44,45,55,56,57,60,61,62,63], three were longitudinal [71,72,75], three were cross-over [66,67,69] and one study had both a longitudinal and cross-sectional design [79]. For social interaction, six articles were of cross-sectional design [43,46,47,56,57,61] and one article was longitudinal [73]. In environmental factors, 11 articles were cross-sectional [48,49,50,51,52,53,54,56,57,64,65], two were longitudinal [74,76] and one was cross-over [68].

### 3.2. Physical Activity

#### 3.2.1. General Description

The association between physical activity and exposure to blue spaces was examined in 18 papers [29,32,33,34,35,36,37,38,39,48,56,57,58,59,60,72,78,79]. Fourteen papers reported blue spaces to have at least one positive association on physical activity, such as a higher volume of physical activity [70], a lower probability of inactivity [78] and more intense physical activity (more Moderate to Vigorous Physical Activity (MVPA)) [32]. Living closer to blue space or in an area with more blue space or more blue space surface was generally reported to positively influence physical activity compared to living further away or in areas with less blue space surface, in adult populations. Grow et al. [33] also found a negative association between proximity to blue space and walking for children, while Wang, Ettema and Helbich [59] found negative associations between the amount of blue space within an area and transportation/recreational walking in adults. Only four studies reported non-statistically significant associations [31,55,56,57].

#### 3.2.2. Physical Activity Measurement Types

Out of the 14 papers showing an association between blue spaces and physical activity, 10 papers showed a positive impact either on walking or MVPA [32,33,34,36,37,38,47,70,77,78]. Of these, Grow et al. [33] also found a negative association between walking and blue space for children. Wang, Ettema and Helbich [59] found negative associations between transportation and recreational walking and blue space, for adults. Pasanen et al. [35] used a more inclusive physical activity indicator, namely “on-land outdoor physical activity”. They reported more “on-land outdoor physical activity” for closer proximity to blue space [35]. Garrett et al. [28] investigated the relationship between “meeting the physical activity guidelines” and blue space. They found higher odds of “meeting the physical activity guidelines” for more freshwater coverage and closer distance to blue space. Apart from MVPA, Jansen et al. [80], also used light physical activity (LPA) as a physical activity indicator in their study, finding a positive association between blue space and LPA.

Cycling was used as a physical activity indicator in two of the 14 papers showing an association between blue spaces and physical activity [33,34].

#### 3.2.3. Quality Assessment

In general, all 18 physical activity related papers were of very good quality, with an average quality score of 88.12%. Papers were downgraded mainly due to insufficient justification of their methods of blue space measurement and the need for a more detailed explanation of their statistical analyses. Quality scores are provided in Supplementary File/Table S4.

#### 3.2.4. Meta-Analyses

There was sufficient data to meta-analyse the effect of distance between blue space and dwelling/neighbourhood and the effect of the amount of blue space on physical activity.

**Figure 3:**
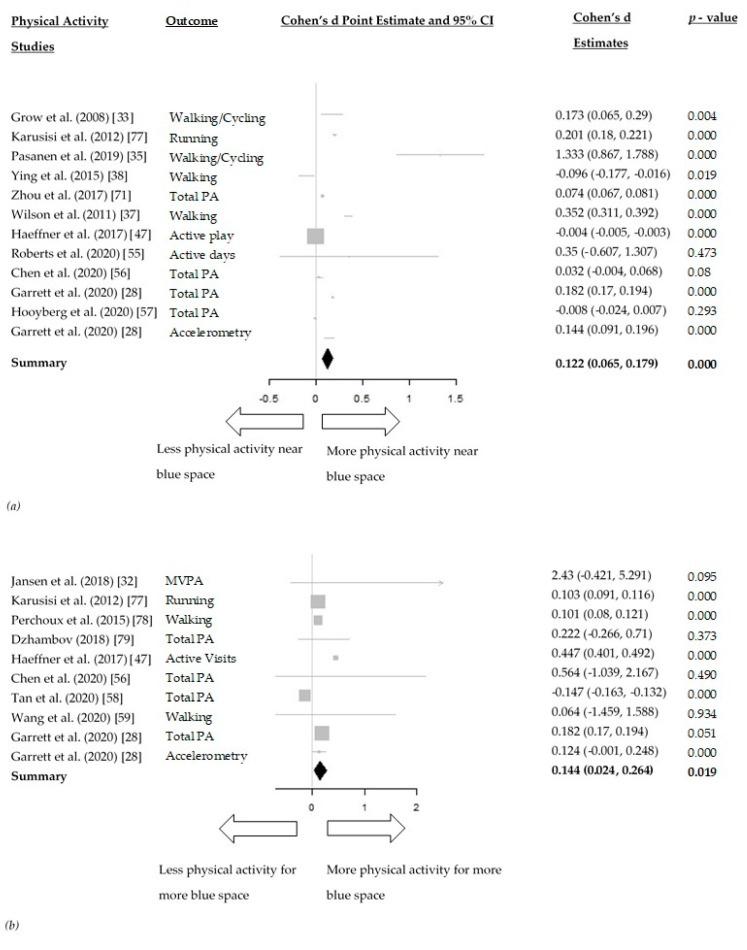
(**a**) Forest plot of the relationships between distance to blue space and physical activity; (**b**) Forest plot of the relationships between the amount of blue space around a certain geographical area and physical activity.

The meta-analysis between distance to blue space and physical activity included 11 studies [28,33,35,37,38,47,55,56,57,70,77]. In the random-effects model, living closer to blue space was associated with statistically significant higher physical activity levels (Cohen d = 0.122, 95% CI: 0.065, 0.179) (Figure 3a). The effect size was low.

The meta-analysis between the amount of blue space and physical activity included nine studies [28,32,47,56,58,59,77,78,79]. A larger amount of blue space within a geographical area was statistically significantly associated with higher physical activity levels (Cohen d = 0.144, 95%CI: 0.024, 0.264) (Figure 3b). The effect size was low and similar to the effect between distance to blue space and physical activity.

Considerable heterogeneity was present in both models with an I^2^ of 99.49% and 99.34% for the association between distance to blue space and amount of blue space, respectively, with physical activity.

### 3.3. Restoration

#### 3.3.1. General Description

The association between exposure to blue spaces and restoration was explored in 21 articles [39,40,41,42,43,44,45,55,56,57,60,61,62,63,66,67,69,71,72,75,79]. 18 articles reported statistically significant effects of blue spaces on restoration [40,41,42,44,45,55,56,60,61,62,63,66,67,69,71,72,75,79], while three articles [39,43,57] did not find an association. Living closer to blue space or in an area with more blue space or more blue space surface was generally reported to positively influence restoration compared to living further away or in an area with less blue space or blue space surface, in adult populations. Contrastingly, in children, Huynh et al. [40], did not find an association of blue spaces with restoration.

More specifically, seven studies found a beneficial effect of blue space availability or visibility on stress or psychological distress [41,43,55,56,63,66,71], while four articles used mental or emotional wellbeing as a restoration indicator and also found a beneficial effect [40,60,66,71]. Positive effects of blue space availability or visibility on anxiety or mood disorders were reported in one study [42]. Pearson et al. [42] suggested that proximity to Great Lakes had a positive effect on mood disorders but proximity to inland lakes had a negative effect. Positive effects of blue space availability or visibility were also found for other measures of restoration, such as attention restoration [71], self-reported history of depression [39], General Health Questionnaire (GHQ-12) scores [61,79], self-reported experienced restoration [62], Short Form 36 health survey (SF-36) scores [44,56] self-reported negative feelings [67], feelings of fascination or “being away” [63] state body shape, appearance and weight satisfaction [75], Patient Health Questionnaire (PHQ-9) [55], Wellbeing Index (WHO-5) [60,69], self-reported life satisfaction [69] and encounters of daily happy moments [72]. Blue space availability/visibility was also found to reduce major depressive disorders, by Rugel et al. [43].

#### 3.3.2. Quality Assessment

Overall, the 21 restoration–related articles were judged as of very good quality with an average quality score of 88.77%. The main reasons for lower quality scores were the insufficient justification of methods of blue space measurements and the lack of a detailed description of input variables. Quality scores are provided in Supplementary File/Table S4.

#### 3.3.3. Meta-Analyses

Sufficient data were available to conduct a meta-analysis of the association between distance to blue space and restoration, amount of blue space within a geographical area and restoration, contact with blue space and restoration.

**Figure 4:**
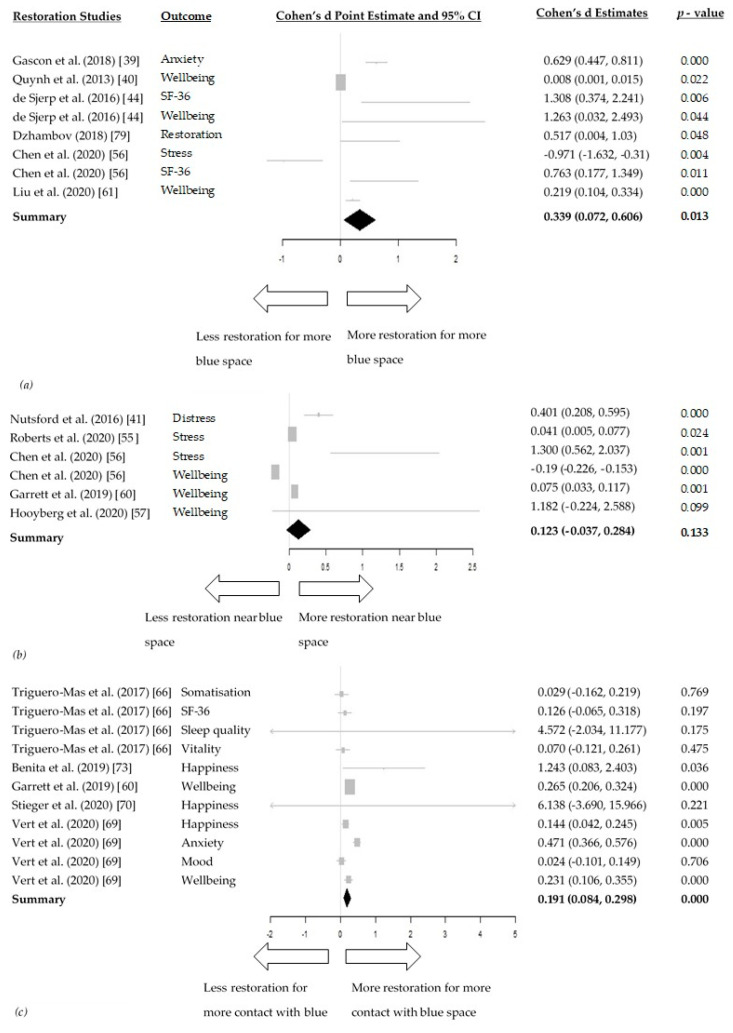
(**a**) Forest plot of the relationship between the amount of blue space within a geographical area and restoration; (**b**) Forest plot of the relationships between distance to blue space and restoration; (**c**) Forest plot of the relationship between contact with blue space and restoration.

For the effect of amount of blue space within a geographical area on restoration the meta–analysis included six studies [39,40,44,56,61,79] pulling together the effects of blue space on five markers of restoration. For the effects of distance to blue space on restoration and contact with blue space on restoration, both meta-analyses included five studies [41,55,56,57,60] and [60,66,69,72,75] respectively. In the random-effects models, the increase of amount of blue space within a geographical area showed a small to moderate, but positive association with improved markers of restoration (Cohen d = 0.339, 95% CI: 0.072, 0.606, I^2^ = 91.97%) (Figure 4a). Having blue space closer to a dwelling/neighbourhood was not associated with higher restoration (Cohen d = 0.123, 95% CI: −0.037, 0.284, I^2^ = 96.60%) (Figure 4b) and being in more contact with blue space was associated with more restoration (Cohen d = 0.191, 95% CI: 0.084, 0.298, I^2^ = 79.50%) (Figure 4c). High heterogeneity was present in all three meta-analyses for restoration.

### 3.4. Social Interaction

#### 3.4.1. General Description

Social interaction was associated with exposure to blue spaces in seven articles [43,46,47,56,57,61,73]. Generally, there was evidence that increasing contact with blue space, decreasing distance between dwellings/neighbourhoods and increasing the amount of blue within a geographical area could improve neighbourhood perception and social interaction, but that this may depend on the scale of each blue space setting.

More specifically, De Bell et al. [46] found that blue space exposure was associated with increased time with family or friends. This benefit, deriving from blue space exposure, appeared smaller than the positive effect of blue space exposure on psychological wellbeing, among people aged between 25 and 65. Hipp et al. [73] also found positive effects of blue space exposure on social interaction, as closer proximity to blue space increased the neighbouring, cohesion and attachment indices of the study’s population. Other markers of social interaction, such as sense of community [81], neighbourhood attachment, community participation and social cohesion [61], were also found to benefit from blue space. On the contrary, increasing a river’s length in a neighbourhood was found to be associated with lower neighbouring cohesion and attachment indices [73]. More amount of blue space within a geographical area was also found to decrease neighbourly interaction [61].

#### 3.4.2. Quality Assessment

The social interaction related articles were judged to be of very good quality with an average quality score of 88.95%. The main reason for lower quality scores was insufficient details when reporting results. Quality scores are provided in Supplementary File/Table S4.

#### 3.4.3. Meta-Analyses

There were sufficient data to meta-analyse the effect of amount of blue space within a geographical area on social interaction and the effect of distance to blue space on social interaction.

**Figure 5:**
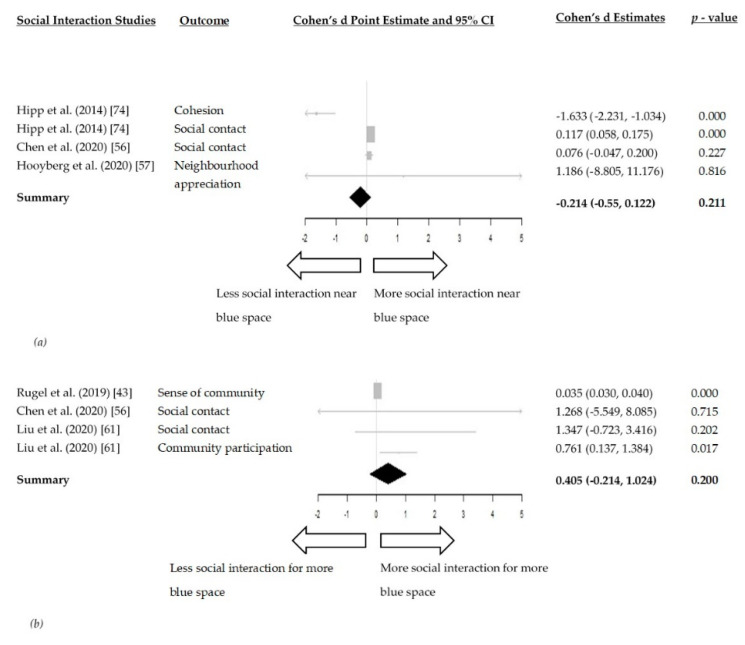
(**a**) Forest plot of the relationship between distance to blue space and social interaction; (**b**) Forest plot of the relationship between amount of blue space within a geographical area and social interaction.

The meta-analysis between distance to blue space and social interaction included three studies [56,57,73]. In the random-effects model, living closer to blue space was not associated with higher levels of social interaction (Cohen d = −0.214, 95% CI: −0.55, 0.122, I^2^ = 90.81%) (Figure 5a).

The meta-analysis of the effect of the amount of blue space within a geographical area and social interaction included three studies [43,56,61]. The amount of blue space within a geographical area was not associated with higher levels of social interaction (Cohen d = 0.405, 95% CI: −0.214, 1.024, I^2^ = 56.41%) (Figure 5b).

### 3.5. Environmental Factors

#### 3.5.1. General Description

Environmental factors were found to be associated with the presence of blue spaces in 14 articles [48,49,50,51,52,53,54,56,57,64,65,68,74,76]. Generally, the presence of blue space in a geographical area was found to positively affect environmental factors, such as lower heat stress index, decreased land surface temperature, higher self-perceived ecological quality of an area and improved air quality mainly through PM2.5 concentrations. Negative effects of blue space presence on environmental factors were found in two studies, regarding increased anthrophony or reduced biophony in a park area [50], increased disease transmission in a developing country context [52] and increased air pollution (PM10) due to river dust [76].

Five articles found a positive effect of blue spaces on temperature [48,49,53,64,65], four articles found a beneficial effect of blue space presence on air quality through lower PM2.5 concentrations [56,57,68,74], two articles presented a positive effect of blue space presence on ecological quality [51,54], one article found an association between increased disease transmission and blue space proximity [52], one article presented increased anthrophony and decreased biophony near a park area [50] and one article found negative effects of blue space on air quality due to increased river dust [76]. Several measures of environmental factors were obtained for each of the environmental factors. Specifically, temperature changes were approached through land surface temperature measurements by Burkart et al. [48] and Wu et al. [65]. Klok et al. [49] operationalised temperature through physiological equivalent temperature, while Saaroni and Ziv [53] used the heat stress index. Another measure of temperature, namely thermal sensation vote, was used by Lehnert et al. [64]. Air quality improvements were described by lower PM2.5 concentrations by Liu et al. [74] and McNabola, Broderick and Gill [68]. Chen et al. [56] approached air pollution through an air quality index obtained from annual data for Guangzhou, China, in 2018. Miró et al. [51] operationalised ecological quality by species richness, while Smith and Moore [54] used self–perceived ecological quality. Environmental noise was measured through anthrophony and biophony by Kuehne, Padgham and Olden [50] and disease transmission was approached through parasitaemia due to increased rainfall and closer proximity to blue spaces in the article by Raso et al. [52]. The negative impact of blue space on air quality through river dust was approached through PM10 air pollution by Chen et al. [76], while the same measure (PM10 air pollution) showed positive effects in a study by Hooyberg et al. [57]. Data were not sufficient for a meta-analysis to be conducted.

#### 3.5.2. Quality Assessment

Overall, the 14 articles presenting an association between blue space presence and environmental factors were judged as of good quality with an average quality score of 80.43%. The main reasons for lower quality scores were insufficient details in the presentation of results, lack of confounding variables and reported statistics, complex study design and lack of a detailed justification of the methods. Quality scores are provided in Supplementary File/Table S4.

## 4. Discussion

This review aimed to synthesise the existing evidence about the mechanisms that mediate the impact of blue space on health, specifically physical activity, restoration, social interaction and environmental factors, and quantify these pathways. Fifty studies were included in our systematic review, of which 27 studies provided data for meta-analyses.

Overall, there was evidence to indicate that blue space increases physical activity, enhances restoration and improves environmental factors. Blue space may also have a beneficial effect on social interaction, but the evidence was mixed and further research is needed on this hypothesised pathway. Thus, three of the four hypothesised pathways (physical activity, restoration, environmental factors) are supported by empirical evidence, while findings for social interaction are inconclusive.

Interestingly, the beneficial effects of blue space on physical activity were almost equally obtained through a shorter distance of someone’s residence to blue space (Cohen d = 0.122, 95% CI: 0.065, 0.179) and a greater amount of blue space around a geographical area (Cohen d = 0.144, 95% CI: 0.024, 0.264). Empirical evidence, therefore, suggests that the development of blue space within shorter distances to residences and increasing the amount of blue space within neighbourhoods could significantly benefit health through the mediating pathway of physical activity.

Our meta-analyses indicated that the blue space benefits on restoration where mainly acquired through a higher amount of blue space within a geographical area (Cohen d = 0.339, 95% CI: 0.072, 0.606), compared to increased contact with blue space (Cohen d = 0.191, 95% CI: 0.084, 0.298). Intriguingly, the increase of amount of blue space within a geographical area was found to be the highest among all mediating pathways and exposures. This evidence, therefore, suggests that developing more blue spaces within neighbourhoods could primarily benefit the restorative character of an area. Living closer to blue space was not found to significantly affect restoration (Cohen d = 0.123, 95% CI: −0.037, 0.284). While urbanicity has been found to increase mental disorders through social stress [82], we propose that creating more blue spaces and promoting contact with them can be used to reverse this effect and ameliorate urban living. The aesthetic nature of blue space may also contribute to its beneficial effects on restoration [10].

Our systematic review suggests that several environmental phenomena, such as heat stress and low air quality can benefit from the development of blue space in urban settings. The evidence base was small, heterogeneous in terms of environmental definitions and measures (precluding a meta-analysis) and mainly focused on heat-related and air quality effects. The beneficial effect of blue space on other environmental factors, such as environmental noise, ecological quality and biodiversity, was insufficiently investigated. A better understanding of blue space effects on environmental factors is necessary for it to be used towards microclimate regulation and therefore further research is conducted around this mediating pathway.

Research around the relationship between blue space and social interaction is still in its infancy and evidence was mixed. Our systematic review, therefore, presented contrasting evidence for this mediating pathway. Our meta-analyses did not find significant beneficial effects of living closer to blue space (Cohen d = −0.214, 95% CI: −0.55, 0.122) or having more blue space within a geographical area (Cohen d = 0.405, 95% CI: −0.214, 1.024) on social interaction.

Findings of this systematic review and meta-analysis are consistent with the existing literature on the salutogenic benefits of blue spaces [8,15,83]. Our review compliments the existing literature by taking a more in-depth look at the mechanisms and mediating pathways between blue space and health. Our findings are also consistent with reported green space benefits, such as increased physical activity [84], increased restoration (e.g., through lower stress levels) [85,86] and improved environmental factors (heat stress) [87]. We therefore suggest that blue space can act as an equally beneficial asset in urban settings, compared to green space, and should be given more attention in future research.

Having reviewed the empirical evidence on the beneficial effect of blue space on the four hypothesised pathways, we suggest that future research should focus on clarifying which particular blue space features have the strongest effect on each mediating pathway. Simultaneously, there is a clear need for more and higher quality research around the effect of blue space on social interaction; research on this lacked consistency and results were found to be inconclusive. This review further highlighted that most studies on the relationship between blue space and environmental factors lacked comparability in terms of outcome measures and failed to account for key confounding factors. Our review highlights the inclusion of more confounding variables in environmental health research, a better definition of blue space elements and the adoption of widely used measures. This is in line with recommendations for future research by Yu et al. [88], who highlighted the complexity and uncertainties of the relationship between blue space and temperature variations. Finally, the majority of included studies were cross–sectional, highlighting the need for more longitudinal research to allow for causality estimation.

### 4.1. Strength and Limitations

This was a comprehensive review evaluating 50 studies. Our review followed the PRISMA guidelines and had a published protocol. It has also followed guidelines for the composition of systematic reviews in research [21]. The abstract and full-text screening was conducted by independent reviewers. The inclusiveness and design of this review can therefore be considered of high quality.

Interestingly, the search for our systematic review indicated that frequently water bodies are included in existing green spaces categories. Blue spaces are often not separated from green spaces, and simply treated as a category of green spaces. For instance, in a study by Sikorska et al. [89], water bodies were one of the types which could guarantee improved access to urban green spaces but they were treated as one of the categories of greenery. Thus, effect sizes for blue spaces might have been underestimated.

As in the case of green spaces [90], accessibility and availability barriers play a significant role in the use of blue spaces. Thus, measures of exposure to blue space, such as distance to blue space or amount of blue space, reflect presence of blue space but do not entirely explain real time of effective engagement with them. This is therefore another limitation of this study and further research should be conducted on the effect of accessibility barriers of blue spaces.

High heterogeneity was found in six of our seven meta-analyses conducted for distance to blue space and physical activity, amount of blue space and physical activity, amount of blue space and restoration, distance to blue space and restoration, contact with blue space and restoration and distance to blue space and social interaction. Their I^2^s ranged between 79.50% and 99.49%. The meta-analysis between amount of blue space and social interaction had a comparatively lower I^2^ of 56.41%. Potential sources of heterogeneity were the wide range of physical activity and restoration indicators used, differences between study designs and the lack of universal measures of blue space exposure. Thus, high heterogeneity should be expected in this type of research. Results of meta-analyses with high heterogeneity should be considered with caution [91]. It is likely that effect size might have been underestimated.

### 4.2. Quality Assessment

The overall quality of the articles included in this review was judged to be very good, as no mediating/causal pathway had an average article quality lower than 70%. These quality ratings show promise for research in this sector, as studies have been well-designed.

### 4.3. Study Design

The majority of the articles included in this review had a cross-sectional design (70.00%), followed by longitudinal (14.00%) and cross-over (8.00%) studies. This, together with the recognition that blue space effects are not immediate, but develop over time, may explain why relationships found in our meta-analyses are weak. According to Rindfleisch et al. [92], a longitudinal study design is more appropriate when looking at events or variables with a clear temporal nature. This may be the case of blue space effects and more longitudinal studies are therefore necessary in the future.

### 4.4. Blue Space Exposure

Articles included in this review measured exposure to blue space in several ways, which subsequently created compatibility difficulties between articles for our meta-analyses to be conducted. As explained above, the lack of universal measures of blue space exposure led to high heterogeneity in all three meta-analyses. Seventeen different measures of blue space exposure were used in the existing literature, namely 100 m, 300 m and 500 m buffers around residencies, coastal proximity of residencies, access to parks/blue space, the proportion of visible water surfaces, frequency of use using GPS devices, road network access/distance, 1 km circular buffers around residencies, GPS mapping of people’s activities near water, self–assessed distance to blue space, self–assessed use of blue space over time, proportion of blue space per municipality using GIS technology, 5 km buffer around schools, proportion of postcode occupied by blue space, self—assessed visual exposure and participation in activities around blue space, distance of blue space to a neighbourhood, self-perceived distance to blue space, normalised difference water index (NDWI), ratio of “blueness” in street view images, minimal distance to water body and polygons using satellite imaging. As a result, a meta-analysis for each health mediator could not be performed, as very few articles shared the same definition or measurement of blue space exposure. An internationally recognised definition and measurement tool for blue space exposure would improve comparability and allow for quantification and calculation of aggregate effects.

### 4.5. Measuring Impact on Health Mediators

Articles related to physical activity used 13 different assessment methods, namely moderate to vigorous physical activity (MVPA), light physical activity (LPA), self-assessed time spent exercising around a blue space per day, self-perceived time spent being active, jogging, recreational walking, watersports, on–land physical activity within 5 km of a blue space, times of walking to work per week, walking more or less than 300 min within a 6.6 km distance to blue space, meeting of physical activity guidelines, health-enhancing energy expenditure per week and percentage of physically active neighbours.

Restoration studies mostly used subjective measures, while few studies had more experimental methods. There were 23 different restoration indicators; self-assessed stress, self–assessed attention restoration, self-assessed mental wellbeing, GHQ-12 scores, SF-36 scores, PHQ-9 scores, self–assessed restorative quality, self–reported history of depression, self-reported events of positive/negative mood, self-reported psychological distress, K10 scores, anxiety or mood disorder, history of major depressive disorder, history of use of antidepressants, visits to mental health specialists, stress measurements with EEG devices, self-perceived life satisfaction, WHO-5 wellbeing index, state physical appearance, state body shape, state weight satisfaction, recalled wellbeing and number of self-reported happy moments among children.

Articles looking at social interaction used seven different measures, namely self–reported number of visits of blue space with friends, self–reported familiarity with neighbourhood, self–reported sense of neighbouring, self–perceived attachment to neighbourhood, self-reported community participation, self-perceived social appreciation and self –perceived cohesion of neighbourhood. Interestingly, all social interaction measures were self–reported, which might include some personal predisposition and therefore bias. It is as a result, needed for a more neutral tool for social interaction to be established and adopted in future research.

Considerable variability was also found in measures of environmental factors as relevant articles used six different environmental measures; air quality (PM2.5), humidity, heat stress index, ecological quality, noise and land surface temperature. Given the nature of the field, no measure was self–reported and all measures were taken using electronic devices or GIS tools. This provides us with robust measurements and any bias should be attributed to malfunction of sensors or range issues.

This prominent variability of tools/types within health mediators led to a high degree of methodological heterogeneity in our meta–analyses. On the other hand, the large number of several physical activity, restoration, social interaction measures, as well as environmental factors, prove the complexity of the field and the need for more multidisciplinary research and cooperation.

### 4.6. Blue Space Types

Blue space benefits and use are not equally distributed among people of different age and socio-economic status [46]. Issues of equality may also exist between different types and quality of blue space. Indeed, among the 50 articles included in this review, only eight looked at a particular blue space type or considered blue space properties, such as size, length or position. Specifically, Pearson et al. [42] found that Great Lakes had a larger and positive effect on anxiety/mood disorder hospitalisations than inland lakes. Furthermore, Hipp et al. [73] found that increasing the length of a river had a negative effect on neighbouring, neighbourhood attachment and neighbourhood cohesion. Looking at three blue spaces with different urbanisation levels (low, medium, high), the location of a blue space was considered by Liu et al. [74]. Interestingly, they found that dry disposition was higher at a greater urbanisation level, which further supports the notion that blue space should be a priority for urban planners especially in metropolitan cities. Miró et al. [51] looked at sustainable drainage systems (SuDS) and suggested that SuDS created as ponds offer a higher ecological quality than SuDS created as swales or detention basins. This finding is a logical continuation of Grizzetti et al. [93] that highlight the positive relationship between the ecological condition and potential recreational capacity of aquatic environments. The temperature changes of a certain pond in Israel at different times and sides were investigated by Saaroni and Ziv [53]. They found that downwind sides of the pond had significantly lower temperatures, lower heat stress index and more relative humidity than upwind sides [53]. This suggests that specific weather features should be considered when deciding on the location of new urban blue spaces. Lehnert et al. [64] looked at fountains, finding a beneficial effect on thermal sensation vote. Respectively, the positive effect of two courtyard pools was investigated by Amirbeiki et al. [63], reporting a beneficial effect on students’ pleasantness, refreshment and relaxation. The negative effects of two specific rivers on air quality through river dust were examined by Chen et al. [76], finding that, based on the position of the rivers, weak northeast monsoons cause the highest health risk in the area.

This review found a small proportion (16.00%) of articles looking at specific blue features. The amount of knowledge derived from the above eight articles looking at specific blue space features highlights the need for more research in this area and emphasises the need to consider blue space independently from other outdoor environments.

### 4.7. Confounders

The confounding effects of variables such as age, gender, socio-economic status and education were considered in most of the studies included in this review. Indeed, several articles have highlighted the fact that blue space use is dependent on age, with potential differences between children and adults in their interactions with blue space [33,40]. De Bell et al. [46] found that women appreciated nature more than men. People with better education were more likely to access blue spaces, while those with lower income faced issues of blue space access or availability [94]. It follows that the indirect impact of age, gender, socio-economic status and education should continue to be considered in future research. Other confounding factors, including average time spent at home a day, presence of chronic disease, body mass index (BMI), ownership of dog and energy expenditure at work, were considered in some of the articles included in this review (Table 1).

### 4.8. Comparison to Existing Literature

To our knowledge, two other systematic reviews and a narrative overview have been conducted around the salutogenic effect of blue space [8,15,83]. The systematic review by Gascon et al. [8] explored the relationship between outdoor blue spaces, health and wellbeing. They included 35 studies and found that higher levels of exposure to outdoor blue spaces were associated with better health and wellbeing, while they also highlighted the need for more longitudinal studies in future research [8]. Kabisch, van den Bosch and Lafortezza [83] explored the effects of green and blue spaces on health, among children and the elderly. Compared to the aforementioned systematic review, these authors included fewer studies in their systematic review (27 studies). They found a positive trend in the relationship between green and blue spaces and health, but results appeared inconclusive, lacked consistency and depended on socio-economic factors [83]. Within their research, green and blue spaces were combined under the umbrella term ‘nature-based solutions’, and so the health impacts of blue spaces were not considered independently. The narrative overview by White et al. [15] explored the potential benefits of blue space on both human and planetary health and wellbeing through mediating pathways, such as physical activity levels, urban temperature variations, social relations and stress. They developed a framework highlighting the existence of the mediating pathways used in our systematic review and meta-analysis.

Our review looked deeper into the relationships between blue space and health, including only articles with blue space as an independent environment. This review explored the effect of blue space on the health “mediators”, such as physical activity, restoration, social interaction and environmental factors. To our knowledge, no other systematic review has looked at health mediators, and this review can therefore be considered a logical continuation of the existing narrative overview by White et al. [15] and systematic reviews by Gascon et al. [8] and Kabisch, van den Bosch and Lafortezza [83].

## 5. Conclusions

This systematic review and meta-analysis has summarised and quantified evidence about mechanisms of the salutogenic effect of blue space on health. We found empirical evidence to support three hypothesised pathways. Blue spaces promote physical activity and increase restoration. They also improve environmental factors, however more research is necessary for meta-analyses to be conducted on this third mediating pathway. The evidence about the role of social interaction is ambiguous. Findings for blue spaces are consistent with reported green space benefits. Considering that most cities in the world are built around blue spaces such as coasts, lakes and rivers, blue spaces are potentially valuable public health assets, which may help reduce the health risk factors associated with increased urbanisation.

## Figures and Tables

**Table 1:** Characteristics of included studies.

Study	Country	Study Design	Population	N	Age Mean (SD)/Age Structure of Sample	Method of Blue Space Measurement	Exposure	Included in Meta-Analysis?	Initial (Prior to Conversion) Measure of Association Reported Included in Meta-Analysis:	Mediating/Causal Pathway	Confounders	Results
Arbillaga-Etxarri et al., 2017 [31]	Spain	Cross-sectional	COPD patients	410	69 (9)	Presence of blue space within 300 m of residence	Amount of blue space	No	-	Physical activity	age, sex, socio-economic status, dyspnea, exercise capacity, anxiety	No significant association between physical activity and proximity to green and blue spaces. Dog walking and grandparenting were associated with an increase both in time in moderate to vigorous physical activity (MVPA) (18 min/day and 9 min/day, respectively) and in physical activity intensity (76 VMU/min and 59 VMUs/min, respectively)
Jansen et al., 2018 [32]	The Netherlands	Cross-sectional	Adults 45–65, living in Rotterdam and Maastricht	222	56.8 (6.1)	Residences, roads, shopping facilities and hospitality industry (e.g., supermarkets, hotels), public social–cultural facilities (i.e., educational institutes, hospitals), sports terrain (e.g., football fields, swimming pool), recreational area (e.g., picnic places, zoos), city green (e.g., city parks, allotments), larger green (e.g., forests, moorlands) and blue space (e.g., rivers, lakes), within 800 m and 1600 m proximity	Amount of blue space	Yes	Beta	Physical activity	-	More MVPA for more blue space
Grow et al., 2008 [33]	US	Cross-sectional	Children and Adolescents in Boston, Cincinnati and San Diego, US	Children: 87, Adolescents: 124	Children: 7.6 (1.7), Adolescents: 14.4 (1.7)	Frequency of use of one of the following: “indoor recreation or exercise facility (public or private),” “swimming pool,” “school recreation facilities open to the public,” “small public park,” “large public park,” “beach, lake, river or creek,” “bike/hiking/walking trails	Distance to blue space	Yes	Risk ratio (RR)	Physical activity	Proximity to facilities, demographic factors (driver’s license, city, race, parent education, Hispanic ethnicity, gender)	Lower chances for biking/walking near blue space (Children), Higher chances for biking/walking near blue space (Adolescents)
Jansen et al., 2017 [34]	The Netherlands	Cross-sectional	Adults aged 45–65 years in The Netherlands	279	57.1 (10.9)	Blue space (e.g., lakes, rivers, water in parks, seas)	Amount of blue space	No	-	Physical activity	Gender, age, health status, BMI, education, employment, ethnicity, car ownership, having children, having a dog, having a garden, and city (Rotterdam vs. Maastricht)	Increased light physical activity (LPA) and MVPA within 150 m of setting
Karusisi et al., 2012 [77]	France	Cohort	Adults aged between 30 and 79 years, in France	7290	Age (years): 30–44 → Men: 36.43%, Women: 33.61%, Age (years) 45–59 → Men: 43.28%, Women: 38.54%, Age (years) 60–79 → Men: 20.29%, Women: 27.84%	Presence of blue/green space with 1 km radius circular buffers centred on each participant’s residence.	Both amount of blue space and distance to blue space	Yes	Risk Ratio (RR)	Physical activity	Age, sex, individual education, marital status, occupation, household income, home ownership, perceived financial strain, Human Development Index (HDI) based on country of birth, energy expenditure at work over the previous week	More chances of jogging within rather than outside neighbourhood for both more area covered with water and closer distance
Pasanen et al., 2019 [35]	England, UK	Cross-sectional	Adults in England	21,097	16–24 -> 10.85%, 25–34 -> 14.34%, 35–44 -> 17.52%, 45–54 -> 16.71%, 55–64 -> 16.51%, 65–74 -> 13.29%, ≥75 -> 10.77	Coastal proximity (0–1 km, > 1–5 km, > 5–20 km, > 20–50 km, and > 50 km), freshwater coverage (absence or presence of freshwater in the Lower-layer Super Output Area (LSOA))	Distance to blue space	Yes	Beta	Physical activity	Urban/rural status (rural including towns, fringes, villages, hamlets, or isolated dwellings), deprivation (quintile of the Index of Multiple Deprivation), age, sex, education, marital status, annual household income, unemployed/employed, economically inactive (retired or stay-at-home parent), car availability, number of children/infants, long-term limiting illness, analyses for year (2008 or 2012)	More walking for closer to blue space
Perchoux et al., 2015 [78]	France	Cohort	All people in France	4365	53 (-)	Presence of a lake or waterways determined from the 2003 IAU-IDF land use database in each area (residential space, workspace, service space, recreational space, social space, street network).	Amount of blue space	Yes	Odds Ratio (OR)	Physical activity	Age, sex, individual education, employment status, household income, marital status, living with at least one child under the age of 14	Decreased odds of not doing any recreational walking for more blue space with a 500 m radius of the setting
Völker et al., 2018 [36]	Germany	Cross-sectional	Urban residents in Bielefeld and Gelsenkirchen, Germany	1041	Age mean from both areas: 51.5 (-), Bielefeld: 50.93 (-), Gelsenkirchen: 52.38 (-)	Questionnaires of “How quickly can you reach a body of water from your home by foot?” and “What kind of body of water is this?”. Area level sources.	Distance to blue space	No	-	Physical activity	Green space, age, gender, education, qualifications, net household income, education index	More frequent use of blue space when located within a 5-min walk in both areas
Wilson et al., 2011 [37]	Australia	Cross-sectional	People in Brisbane	10,286	40–44 → 2088 (20.3%), 45–49 → 2264 (22.0), 50–54 → 2136 (20.8), 55–59 → 1965 (19.1), 60–65 → 1833 (17.8)	Network distance to nearest river or coast	Distance to blue space	Yes	Odds Ratio (OR)	Physical activity	Age, sex, education, occupation, living arrangement, household income, neighbourhood-level socio-economic disadvantage	Increased odds of walking near blue space
Ying et al., 2015 [38]	China	Cross-sectional	People in Shanghai, China, aged 46–80	1100 from 80 neighbourhoods	-	Existence of blue space in the 500 m residents’ activity buffer radius	Distance to blue space	Yes	Beta	Physical activity	Age, gender, employment status, education	Decreased number of total steps of walking for increased river proximity
Haeffner et al., 2017 [47]	US	Cross-sectional	People living in urban neighbourhoods in Utah, US	1450 households from 13 neighbourhoods	-	Proximity of a participant’s household to its local waterway. Distance from the respondent’s home to the nearest Access Points (Aps) where they could see and/or spend time near the water. Access to such spaces.	Visit to blue space	No	-	Physical Activity	Respondent’s education, household income, homeowner status, race/ethnicity, children present, length of residency	Increased odds of walking, playing or visiting a blue space for presence of public access point (AP) near someone’s residence.
Zhou et al., 2017 [70]	China	Longitudinal	People in Huainan, China	Health survey n = 3094, interviews n = 42	Interview stage: 55–64 years, n = 21, 65+ years n = 21. Health Survey: 55–64 years → 1177 (38%), 65–88 years → 1925 (62%)	Questionnaire to map older people’s activities and then use of GIS to spot the existence of natural or human-made water bodies	Distance to blue space	Yes	Beta	Physical activity	Gender, age, education, income, overweight and obesity, hypertension, diabetes, hyperlipidemia, cardiovascular conditions, liver and biliary system conditions, kidney function	Increased frequency of physical activity per week for closer distance to blue space
Arnberger et al., 2018 [71]	Alpine range from Austria to Switzerland	Longitudinal	Adult people 22 to 36 years old	22	26.7 (4.1)	Existence of meadow/river on site. Visit of 5 different locations in the alpine region	Contact with blue space	No	-	Restoration	-	High restorative potential of mountain rivers. Blue space found to provide health benefits
Dzhambov, 2018 [79]	Bulgaria	Both cross-sectional and longitudinal	Students between 18 and 35 years old, in Plovdiv, Bulgaria	109	21 (3)	Blue space presence in circular buffers of 100 m, 300 m and 500 m around students’ residences	Amount of blue space	Yes	Pearson’s R	Restoration	Age, gender, ethnicity, duration of residence, average time spent at home a day, perceived economic status	Association between restorative quality and blue space
Gascon et al., 2018 [39]	Spain	Cross-sectional	Adults in Barcelona	958	56.5 (-)	Presence of blue spaces of any type and size represented in the map around the residential address (buffers of 100, 300 m and 500 m)	Amount of blue space	Yes	Odds Ratio (OR)	Restoration	Age, gender, educational attainment, marital status, living alone, work category, physical activity, smoking, sleep quality, social support, perceived social support, meditation, caregivers of people with AD or other chronic disease, family history of Alzheimer’s disease (AD) or any other dementia, BMI, air pollution—annual average levels of nitrogen oxides (NO2 and Nox) and particulate matter (PM2.5, PM2.5 absorbance (abs), PM10, and PM coarse).	Lower odds of self-reported history of anxiety, self-reported history of depression, self-reported history of medication with Benzodiazepines, self-reported history of antidepressants use, for more blue space within both a 300 m and 500 m radius.
Huynh et al., 2013 [40]	Canada	Cross-sectional	School students in Canada	17,249 students, 317 schools	≤11 -> 13.8%, 12 -> 20.1%, 13 -> 19.3%, 14 -> 19.2%, 15 -> 19.7%, ≥16 -> 7.8%	Public natural space (green and blue spaces such as parks, wooded areas, and water bodies) within a 5 km radius circular buffer surrounding each school.	Amount of blue space	Yes	Risk Ratio (RR)	Restoration	Socio-economic status, perceived neighbourhood safety (Family affluence scale), neighbourhood aesthetics, neighbourhood SES (median household income, employment rate, percentage of population with greater than high school education, urban/rural geographic location (rural area (<10,000 persons), small city (10,000–99,999 persons), or metropolitan area (>100,000 persons)). Age, gender, ethnicity and urban/rural geographic location as moderators.	Higher chances of positive emotional wellbeing for existence of public natural space within a 5 km radius around school
Nutsford et al., 2016 [41]	New Zeeland	Cross-sectional	People in New Zeeland	442	15–44 yr → Females: 56%, Males: 54%, Total: 55%, 45–64 yr → Females: 32%, Males: 35%, Total: 33%, 65+ yr → Males: 12%, Females: 12%, Total: 12%	Visible blue space within <300 m; 300 m to 3 km; 3–6 km and 6–15 km	Distance to blue space	Yes	Beta	Restoration	Age, sex, personal income, neighbourhood population density, housing quality, crime and deprivation.	More visibility (closer distance) of blue space leads to better scores at the Kessler Psychological Distress scale (K10)
Pearson et al., 2019 [42]	US	Cross-sectional	People in US	30,421	42 (16)	The proportion of a ZIP code occupied by inland lakes; The average Euclidean distance to the nearest blue space boundary, distinguished between inland lakes and Great Lakes.	Amount of blue space	Yes	Beta	Restoration	Median income and population density, age, sex	Decrease of individual-level anxiety/mood disorder hospitalisations for more blue space.
Rugel et al., 2019 [43]	Canada	Cross-sectional	People in Canada	1,930,048	Weighted %: 15–24 years → 17.4%, 25–34 years → 15.3%, 35–44 years → 18.7%, 45–54 years → 19.2%, 55–64 years → 12.8%, 65 and older → 16.5%	Presence of blue space permanent water features such as oceans, lakes, and rivers and intermittent sources such as sloughs and bogs. Visible blue space percentage within a 100-m buffer. Accessible blue space percentage within a 1000-m buffer.	Amount of blue space	Yes	Odds Ratio (OR)	Restoration	Sex, age, race-ethnicity, Provincial household income level (compares the participant with others residing in the same province), highest household education level, household type, household living arrangement (indicates the relationship of the participant with others in the same household), pain health status, urbanicity, population density, walkability.	Stronger sense of community for more blue space
Triguero-Mas et al., 2017 [66]	Spain, England (UK), The Netherlands, Lithuania	Cross-over	People in Spain, England, The Netherlands and Lithuania	Total: 406, Barcelona: 107, Stoke-on-Trent: 90, Doetinchem: 105, Kaunas: 104	Total: 51.00 (26.00), Barcelona: 40.00 (23.00), Stoke-on-Trent: 43.50 (28.75), Doetinchem: 59.00 (16.00), Kaunas: 55.00 (23.25)	Contact with blue space defined as presence/absence within 50 m of each participant’s location point. Residential exposure with a 300 m buffer around residencies.	Contact with blue space	No	-	Restoration	City of residence, age, gender, education, neighbourhood socio-economic status,	Contact with green/blue space led to higher SF-36 mental health scores, 4DSQ scores, Vitality scale scores, number of nights of good sleep.
de Vries et al., 2016 [44]	The Netherlands	Cross-sectional	People in The Netherlands	6621	Age: below 35 N = 1600 (24%) Age: between 35 and 54 N = 3278 (50%)	Blue space availability as percentages of the area within 1 km from one’s home	Amount of blue space	Yes	Odds Ratio (OR) and Beta	Restoration	Gender, age, having a partner, having a child within the household, educational level, having a paid job, household income, urbanicity of the respondent’s neighbourhood, socio-economic status of the neighbourhood (by average residential property value)	Lower odds of anxiety disorder, any mood disorder, substance use disorder, common mental disorder for more blue space within 1 km from someone’s residence. Better self-perceived mental health scores (SF-36), self-perceived general health scores for more blue space within 1 km from someone’s residence.
Triguero-Mas et al., 2015 [45]	Spain	Cross-sectional	People in Catalonia, Spain	8793	48 years	Access to blue spaces	Amount of blue space	Yes	Odds Ratio (OR)	Restoration	Gender, age, education completed, birth place, type of health insurance, marital status, indicators of household based on the occupation of the main person of each household, neighbourhood (the percentage of the population with education higher than secondary in the participant’s census track), socioeconomic status (SES)	Better self-perceived general health for blue space, Better self-perceived social support for more blue space
Reeves et al., 2019 [67]	England, UK	Cross-over	People exposed to wetlands	36	41 (10.28)	Exposure to Wetland, Urban and Control site, London	Contact with blue space	No	-	Restoration	Age, gender, site order, self-reported levels of stress	Lower heart rate for contact with blue space setting compared to urban setting. More positive feelings for blue space setting. Decrease in negative feelings for blue setting.
Benita et al., 2019 [72]	Singapore	Longitudinal	Primary, secondary and junior college students	10,464	-	Parks, water bodies, open spaces as POIs. Visit of a POI during the day. Proximity of POIs to parks, water body and open space/reserve site with a 100 m buffer.	Visit to blue space	No	-	Restoration	Environmental factors (temperature, humidity, noise, daylight), Personal characteristics (age, housing price, social group), day and months	More happy moments among students who visited open spaces.
de Bell et al., 2017 [46]	Britain, Excluding the Isle of Scilly, the Scottish Highlands and Islands	Cross-sectional	People in Britain	1043	6–24 → 11.1%, 25–44 → 32.4%, 45–64 → 33.5%, 65 and over → 23%	Areas such as rivers, canals and lakes and their immediate surroundings, including river paths, canal paths and lakeside walks. Excluded coastal blue spaces such as beaches. Visit to blue spaces.	Visit to blue space	No	-	Social interaction	Age, gender, household composition, socio-economic status, car ownership, health status, urbanicity of the respondents’ dwelling	Higher odds of spending time with family or friends for more visits to blue space.
Hipp et al., 2014 [73]	Australia	Longitudinal	People living in Australia	4351 residents, 146 neighbourhoods	0.512 (0.152)	“Holes” in the social environment: parks and industrial areas “Wedges” in the social environment: Rivers and Highways.	Distance to blue space and size of blue space	No	-	Social Interaction	Residential stability, median income, percent perceived non-Anglo, population density, approximate annual household income, highest level of education, own or rent, length of residence at current address, spoken languages at home other than English, dependent children of respondent, marital status, age, gender, ancestry measures.	Lower neighbouring index for increased size of blue space. Higher neighbourhood attachment index for increased size of blue space. Lower neighbourhood cohesion index for increased size of blue space.
Burkart et al., 2016 [48]	Portugal	Cross-sectional	Elders over 65, in urban areas of Portugal	218,764 deaths from 213 civil parishes	-	Urban blue defines as urban water bodies	Distance to blue space	No	-	Environmental factors (Temperature)	Time trends, age, urban density, socio-economic status	Association between proximity to water and land surface temperature
Klok et al., 2019 [49]	The Netherlands	Cross-sectional	Areas in Amsterdam	21 locations	-	Blue locations—urban areas close to water bodies such as canals, rivers, ponds and fountains in the chosen area.	Amount blue space	No	-	Environmental factors (Temperature)	-	Temperature reduction for presence of blue space
Kuehne et al., 2013 [50]	US	Cross-sectional	People/lakes in Washington state, US	10 lakes	-	Lakes classified as Low (30%), Medium (30–50%), and High (50%) urbanisation	Amount of blue space	No	-	Environmental factors (Environmental noise)	Landscape factors, time period	Presence of public park/lake had a negative effect on biophony. Lakes with higher urbanisation levels led to higher anthrophony and lower biophony.
Liu et al., 2018 [74]	China	Longitudinal	Wetland plots/people in China	3 wetland plots	-	Three wetlands chosen as experimental sites	Amount of blue space	No	-	Environmental factors (Air quality)	-	Greater PM 2.5 removal efficiency for wetlands with higher degree of urbanisation
McNabola et al., 2008 [68]	Ireland	Cross-over	Boardwalks/people in Ireland	1 boardwalk in Dublin	-	Boardwalk next to River Liffey, Ireland.	Distance to blue space	No	-	Environmental factors (Air quality)	Traffic density, temperature, idle time, stability, traffic related turbulence	Pedestrians using boardwalks are less exposed to benzene and PM 2.5 pollution
Miro et al., 2018 [51]	Scotland, UK	Cross-sectional	Sustainable drainage systems (SuDS)/people in Scotland	34 SuDS	-	Areas with Sustainable Drainage Systems (SuDS)	Amount of blue space	No	-	Environmental factors (Ecological quality)	Socio-economic indicators (semidetached houses, terraced houses, three rooms, six rooms, eight rooms, nine rooms, three cars, four cars)	Presence of SuDS leads to higher ecological quality
Raso et al., 2009 [52]	Ivory Coast	Cross-sectional	Children in Ivory Coast	3962	1684 children (42.5%) 6–10 years old, 2278 children (57.5%) aged 11–16 years.	Distance of schools to rivers, using digitised ground maps	Distance to blue space	No	-	Environmental factors (Disease transmission)	Spatial correlation, age, bed net coverage, rainfall during the main malaria transmission season, distance to NDVI for vegetation	Association between proximity to rivers and P. falciparum infections
Saaroni and Ziv, 2003 [53]	Israel	Cross-sectional	Ponds/people in Israel	1 pond in Tel Aviv, Israel	-	A pond of 4 ha. Four stations located 3–5 m from the edge of the pond (north, south, east, west)	Distance to blue space	No	-	Environmental factors (Temperature)	-	Lower temperatures, higher relative humidity and lower heat stress index downwind compared to upwind
Smith and Moore, 2011 [54]	US	Cross-sectional	Recreationists in US	247 Recreationists at Farmington River, 841 Recreationists at Chattooga River	Farmington River: 47.7 (13.8), Chattooga River: 41.0 (11.8)	Farmington River, Chattooga River	Distance to blue space	No	-	Environmental factors (Ecological quality)	Age, gender, income, race, education, trips within the past 12 months, miles travelled from home to river	Decreased self-perceived ecological benefits for increased proximity to river.
Roberts, van Lissa and Helbich, 2021 [55]	The Netherlands	Cross-sectional	People in the The Netherlands	11,505	18–24 years old -> 1301 (11.3), 25–35 years old -> 2143 (18.6), 36–45 years old -> 1979 (17.2), 46–55 years old-> 2817 (24.5), 56–65 years old -> 3265 (28.4)	Self-perceived distance to blue space: “Less than 300 m”, “≥300 m to 1 km”, “≥1–5 km” and “≥5 km or more”	Distance	Y	Beta	Physical activity, Restoration	Age, sex, ethnic origin (Dutch, Western migration background, Non-Western migration background), marital status (married, separated/divorced, widow, never married), education level (low, medium, high), income quintile (1 = lowest quintile, 5 = highest quintile), and household type (single parent, couple without children, couple with children, other household type), urbanity, deprivation, and social fragmentation.	Increased days of being physically active for at least 30 min over the past 7 days for closer distance to blue space (At home group). Decreased days of being physically active for at least 30 min over the past 7 days for closer distance to blue space (Working group). Lower stress for closer distance to blue space (Working and at home groups)
Chen and Yuan, 2020 [56]	Guangzhou, China	Cross-sectional	Elderly individuals in Guangzhou, China	966	69.33 (7.77)	NDWI (Normalised difference water index), distance to nearest water body, proportion of water area in a 1 km buffer zone of neighbourhood boundary, per capita water area	Distance to blue space, Amount of blue space	Y	Beta	Physical activity, restoration, social interaction, environmental factors	Age, gender, educational attainment, marital status, hukou status, monthly household income, employment information	More physical activity for higher proportion of water area, more per capita water area, closer distance to blue space. Less physical activity for more NDWI. Better SF-36, more stress scores for more NDWI. Less stress, better SF-36 scores for closer proximity to blue space. Better SF-36, lower stress scores for more proportion of water area. Worse SF-36 scores, more stress for more per capita water area. More social contact for more NDWI, proportion of water area, closer distance to blue space. Less social contact for more per capita water area. Better air quality for more NDWI, per capita water area, proportion of water area and closer distance to blue space.
Garrett et al., 2020 [28]	England, UK	Cross-sectional, cross-over	Adults in the UK	1774 in accelerometer analysis, 18,447 main analysis	All aged 16+	Residential coastal proximity categorised as <5 km, 5–20 km and >20 km. Percentage freshwater coverage of each LSOA from the CEH Land Cover Map 2007 and categorised as (0%, >0–1%, >1–5%, >5–100%).	Amount of blue space, Distance to blue space	Y	Odds Ratio (OR)	Physical activity	Equivalised household income, (a) area-level—neighbourhood deprivation (LSOA IMD; quintiles; most deprived = reference category); (b) household-level—number of children (none = ref.); access to car/van (has access = ref.); (c) individual-level—age (categorised in 20 year intervals; 16–34 = ref.); sex (female = ref.); highest qualification (none/foreign/other = ref.); employment status (in work/education = ref.); marital status (single = ref.); limiting illness (limiting illness = ref.); BMI (normal weight = ref.); smoking (current smoker = ref.); and (d) year of survey (2008 = ref).	Higher odds of meeting the physical activity guidelines for more freshwater coverage and closer distance to blue space.
Hooyberg et al., 2020 [57]	Belgium	Cross-sectional	People in Belgium	60,939	42.7	Residential proximity calculated as the distance travelled using the fastest driving route from the geographical centre of the residential municipality to the nearest point at the Belgian coast	Distance	Y	Beta	Physical activity, Restoration, Social Interaction	Age (< 20 year, 21–45 year = ref, 46–65 year, > 65 year), sex (male = ref, female), having a chronic disease (yes, no = ref, no answer), BMI (normal weight = ref, underweight, obesity class I, obesity class II, obesity class III), employment status (employed = ref, unemployed), income (quintile 1, quintile 2, quintile 3, quintile 4, quintile 5 = ref, no answer), smoking status (nonsmoker = ref, occasional smoker, daily smoker, no answer) and level of urbanization (urban = ref, sub-urban, rural), year (1997, 2001, 2004 = ref, 2008, 2013) and season (winter = ref, spring, summer, fall), blue space ratio, green space ratio	Lower physical activity for closer distance to blue space. Better GHQ-12 scores for closer distance to blue space.
Tan et al., 2021 [58]	Singapore	Cross-sectional	People in Singapore	1471	All participants aged 17+	Cover of blue space around 250 m, 500 m, 1 km, 1.5 km from home	Amount of blue space	Y	Odds Ratio (OR), Beta	Physical Activity	Age, gender, highest education qualification, ethnicity, housing type, individual income/allowance, occupational status, number of hours spent at home, exercise choice, exercise frequency	Lower overall exercise frequency for more blue space.
Wang, Ettema and Helbich, 2020 [59]	The Netherlands	Cross-sectional	People in the Netherlands	65,785	18–44 years old -> 38.00%, 45–64 years old -> 39.03%, 65+ years old -> 22.97%	Blue space around respondents’ home addresses for buffers with 300, 600, and 1000 m radius.	Amount of blue space	Y	Beta	Physical Activity	Age, level of education, household income, gender, ethnicity, possession of driving license, household composition, number of cars per household, number of e-bikes per household, number of mopeds per household	Less walking for more blue space in weekdays. More recreational walking for more blue space over the weekends.
Liu et al., 2020 [61]	China	Cross-sectional	People in Guangzhou, China	1150	39.553 (11.065)	Ratio of blueness of street view images within a circular buffer of 1500 ms around the geocoded address of the central point for each sampled neighbourhood. 1500 m buffer area based on remote-sensing data from the GlobeLand30 dataset.	Amount of blue space	Y	Beta	Restoration, Social Interaction	Gender, age, educational attainment, marital status, hukou status, employment status, participation in medical insurance and average household income per household member	Better GHQ-12 scores for more blue space. Better neighbourhood attachment, neighbourly interaction, community participation for more blue space.
Stieger, Aichinger and Swami, 2020 [75]	Austria	Longitudinal	People in Austria	107	26.9 (11.2)	Participants described their surroundings. Blue space defined as lake, sea, river, wetlands	Contact with blue space	Y	Beta	Restoration	Age, sex, CNS scores, and NES scores	Higher state body satisfaction, state body shape satisfaction, state physical appearance, happiness for more contact with blue space.
Subiza-Pérez, Vozmediano and San Juan, 2020 [62]	Spain	Cross-sectional	People in Donostia-San Sebastián (Spain)	429	40.72 (17.82)	Participants reported use of blue spaces based on the natural environment scoring tool	Contact with blue space	N	-	Restoration	Gender, age, access, recreational facilities, amenities, natural features, aesthetics non-natural, incivilities, significant natural features, global score, usability, frequency of use (monthly and weekly), time of use, walking, meeting with friends and relatives, practicing physical activity, reading, landscape contemplation, walking the dog, spending time with dependants, sunbathing/enjoying the sun, eating/drinking something, perceived restorativeness, place attachment, place identification	Users of beaches had higher levels of attachment, identification, and experienced restoration than the participants surveyed in urban parks.
Vert et al., 2020 [69]	Spain	Cross-over	People in Spain	59	29 (min = 19, max = 49)	Participants were randomly assigned to settings (blue spaces, urban areas, control room)	Contact with blue space	Y	Incidence Rate Ration (IRR)	Restoration	Gender, age, education, perceived household income, marital status, residential access to natural spaces (blue and/or green), views of blue spaces at work, access to private open space, blue space exposure during childhood, meeting physical activity WHO guidelines, BMI	Better subjective wellbeing, mood, WHO-5 wellbeing index, life satisfaction, eudaimonic wellbeing for more blue space contact.
Amirbeiki and Ghasr, 2020 [63]	Iran	Cross-sectional	Students in Yazd, Iran	81 students	Participants 20 to 31 years old	Courtyards’ water pools	Contact with blue space	N	-	Restoration	Age, sex, year of study, length of visiting the courtyards	Blue space had the most significant influence on feelings of fascination and being away.
Chen et al., 2021 [76]	Taiwan	Longitudinal	People in Taiwan, Estuary areas in Taiwan	2 rivers in Taiwan (Dajia and Da’an)	-	2 rivers	Distance to blue space	N	-	Environmental factors	-	PM10 concentration increases considerably during both wet and dry seasons near the two rivers.
Lehnert et al., 2021 [64]	Czech Republic	Cross-sectional	People in Brno, Olomouc, Ostrava, Plzen,	1522	-	Fountains	Distance to blue space	N	-	Environmental factors	Biometeorological indices, activity, hour of the day	High thermal sensation vote near blue space.
Wu et al., 2019 [65]	Wuhan, China	Cross-sectional	People in Wuhan, China, 51 lakes	51 lakes	-	Normalized difference water index (NDWI)	Amount of blue space	N	-	Environmental factors	-	The cooling effects of blue space are dependent on size and shape. Lower surface temperature for more blue space.
Garrett et al., 2019 [60]	Hong Kong	Cross-sectional	Adults in Hong Kong	1000	80% of respondents were > 50 years old	Incidental exposure: question of “Do you usually pass by/through this [the nearest] blue space when commuting, to or from work/school/other daily activities?”. Intentional exposure: how often participants visited any blue spaces in the last four weeks. Self-reported measure of proximity within 10–15 min walk from participant’s home. Frequency of visit of the closest blue space to participant’s home. Water contact (direct or not contact with water)	Distance to blue space, Contact with blue space	Y	Odds Ratio (OR)	Restoration	District, physical functioning, age, access to garden, occupation, income, sex, meeting recommended physical activity, children living in household, marital status, dog ownership, others on visit to nearest blue space	Higher odds of higher WHO-5 wellbeing index, better recalled wellbeing for closer distance to blue space and more contact with blue space.

## Data Availability

The data that support the findings of this study are available from the corresponding author, upon request.

## Supplementary Materials

The following are available online at https://www.mdpi.com/1660-4601/18/5/2486/s1, Table S1: Search keywords; Table S2: Search strings; Table S3: Inclusion/Exclusion criteria; Table S4: Quality scores.
