# Supplementary material for: Mechanisms of Impact of Blue Spaces on Human Health: A Systematic Literature Review and Meta-Analysis
Source: Int J Environ Res Public Health. 2021 Mar 3;18(5):2486. doi: 10.3390/ijerph18052486 (PMC7967635; doi:10.3390/ijerph18052486)
Supplement: Supplementary file 1 [file ijerph-18-02486-s001.pdf]

International Journal of
Environmental Research and Public Health

MDPI

Supplementary Materials

Table S1: Search Keywords

Main word	Synonyms/Relevant Words/Keywords
Physical Activity	Physical activities, exercise, exercises
Stress	Stresses, physical pressure, mental pressure, anxiety, panic, panics, anxieties, worry, worries, nervousness, apprehension, mental wellbeing, mental well-being, psychological
Sleep/Sleeps	Bedtime
Air pollutant	Air pollutants, air pollution, air quality, particle pollution, particulate matter
Social interaction	Social interactions, social behaviour, social behaviours, social relation/relations, sense of community, social cohesion, social skills, bonding, group cohesiveness, unity, social wellbeing/well-being, socio-economic condition/conditions, socio-economic status
Noise	Noises, sound pollution, sound pollutant/pollutants
Green and Blue space/spaces	Blue space/spaces, canal/canals, water body/bodies, green and blue infrastructure, blue infrastructure, aqueduct/aqueducts, wetland/wetlands, urban waterway/waterways, riparian corridor/corridors, lake/lakes, river/rivers

Table S2: Search Strings

Pubmed	((green[Title/Abstract] AND blue space[Title/Abstract]) OR blue space*[Title/Abstract] OR canal[Title/Abstract] OR water body[Title/Abstract] OR water bodies[Title/Abstract] OR (green[Title/Abstract] AND blue infrastructure[Title/Abstract]) OR blue infrastructure[Title/Abstract] OR (blue[Title/Abstract] AND infrastructures[Title/Abstract]) OR aqueduct[Title/Abstract] OR aqueducts[Title/Abstract] OR wetland[Title/Abstract] OR wetlands[Title/Abstract] OR urban waterway[Title/Abstract] OR (riparian[Title/Abstract] AND waterways[Title/Abstract]) OR riparian corridor[Title/Abstract] OR riparian corridors[Title/Abstract] OR lake[Title/Abstract] OR lakes[Title/Abstract] OR river[Title/Abstract] OR rivers[title/abstract]) AND (physical activity[Title/Abstract] OR physical activities[Title/Abstract] OR exercise[Title/Abstract] OR exercises[Title/Abstract] OR stress[Title/Abstract] OR stresses[Title/Abstract] OR physical pressure[Title/Abstract] OR physical pressures[Title/Abstract] OR mental pressure[Title/Abstract] OR mental pressures[Title/Abstract] OR anxiety[Title/Abstract] OR anxieties[Title/Abstract] OR panic[Title/Abstract] OR panics[Title/Abstract] OR worry[Title/Abstract] OR worries[Title/Abstract] OR nervousness[Title/Abstract] OR apprehension[Title/Abstract] OR wellbeing[Title/Abstract] OR well-being[Title/Abstract] OR psychological[Title/Abstract] OR sleep[Title/Abstract] OR sleeps[Title/Abstract] OR bedtime[Title/Abstract] OR air pollutant[Title/Abstract] OR air pollutants[Title/Abstract] OR air pollution[Title/Abstract] OR air quality*[Title/Abstract] OR particle pollution*[Title/Abstract] OR particulate matter[Title/Abstract] OR social interaction*[Title/Abstract] OR social behaviour*[Title/Abstract] OR social relation*[Title/Abstract] OR sense of

	community[Title/Abstract] OR social cohesion*[Title/Abstract] OR social skill*[Title/Abstract] OR bonding[Title/Abstract] OR group cohesiveness*[Title/Abstract] OR unity[Title/Abstract] OR social wellbeing[Title/Abstract] OR social well-being[Title/Abstract] OR socioeconomic condition[Title/Abstract] OR socioeconomic conditions[Title/Abstract] OR socioeconomic status[Title/Abstract] OR noise pollution[Title/Abstract] OR noise[Title/Abstract] OR noises[Title/Abstract] OR sound pollution[Title/Abstract] OR (sound[Title/Abstract] AND pollutant*[Title/Abstract]))
Scopus	TITLE-ABS-KEY ("green and blue space*" OR "blue space" * OR canal OR water AND body OR water AND bodies OR "green and blue infrastructure" OR "blue infrastructure" OR "blue infrastructures" OR aqueduct OR aqueducts OR wetland OR wetlands OR "urban waterway" OR "riparian waterway" OR "riparian waterways" OR waterway* OR "riparian corridor*" OR lake OR lakes OR river OR rivers) AND ("physical activity" OR "physical activities" OR exercise OR exercises OR stress OR stress OR "physical pressure" OR "physical pressures" OR "mental pressure" OR "mental pressures" OR anxiety OR anxieties OR panic OR panics OR worry OR worries OR nervousness OR apprehension OR " wellbeing" OR " well-being" OR psychological OR sleep OR sleeps OR bedtime OR "air pollutant*" OR "air pollution" OR "air quality" OR "particle pollution" OR "particulate matter" OR "social interaction*" OR "social behaviour*" OR "social relation*" OR "sense of community" OR "social cohesion" OR "social skill*" OR bonding OR "group cohesiveness*" OR unity OR "social wellbeing" OR "social well-being" OR "socio-economic condition" OR "socio-economic status" OR "noise pollution" OR noise OR noises OR "sound pollution" OR "sound pollutant*")
PhycInfo	(ab("green and blue space*" OR ("blue space") OR canal OR canals OR "water body" OR "water bodies" OR "green and blue infrastructure*" OR "blue infrastructure" OR aqueduct OR aqueducts OR wetland OR wetlands OR "urban waterway*" OR ("riparian corridor" OR "riparian corridors") OR lake OR lakes OR river OR rivers) OR ti("green and blue space*" OR ("blue space") OR canal OR canals OR "water body" OR "water bodies" OR "green and blue infrastructure*" OR "blue infrastructure" OR aqueduct OR aqueducts OR wetland OR wetlands OR "urban waterway*" OR ("riparian corridor" OR "riparian corridors") OR lake OR lakes OR river OR rivers)) AND (ab("physical activity" OR "physical activities" OR exercise OR exercises OR stress OR stresses OR "physical pressure" OR "physical pressures" OR "mental pressure" OR "mental pressures" OR anxiety OR anxieties OR worry OR worries OR nervousness OR apprehension OR "wellbeing" OR "well-being" OR psychological OR sleep OR sleeps OR bedtime OR ("air pollutant" OR "air pollutants") OR "air pollution" OR "air quality" OR "particle pollution" OR "particulate matter" OR ("social interaction" OR "social interactionist" OR "social interactions") OR ("social behaviour" OR "social behaviours") OR ("social relation" OR "social relations" OR "social relationship" OR "social relationships") OR "sense of community" OR ("social cohesion") OR "social skills" OR "social skill" OR bonding OR "group

	cohesiveness" OR unity OR "social wellbeing" OR "social well-being" OR ("socioeconomic conditions") OR "socioeconomic status" OR noise OR noises OR "sound pollution" OR "sound pollutant*") OR ti("physical activity" OR "physical activities" OR exercise OR exercises OR stress OR stresses OR "physical pressure" OR "physical pressures" OR "mental pressure" OR "mental pressures" OR anxiety OR anxieties OR worry OR worries OR nervousness OR apprehension OR "wellbeing" OR "well-being" OR psychological OR sleep OR sleeps OR bedtime OR ("air pollutant" OR "air pollutants") OR "air pollution" OR "air quality" OR "particle pollution" OR "particulate matter" OR ("social interaction" OR "social interactionist" OR "social interactions") OR ("social behaviour" OR "social behaviours") OR ("social relation" OR "social relations" OR "social relationship" OR "social relationships") OR "sense of community" OR ("social cohesion") OR "social skills" OR "social skill" OR bonding OR "group cohesiveness" OR unity OR "social wellbeing" OR "social well-being" OR ("socioeconomic conditions") OR "socioeconomic status" OR noise OR noises OR "sound pollution" OR "sound pollutant*"))
Web of Science	(TI=(("green and blue space*" OR "blue space" OR canal OR canals OR "water body" OR "water bodies" OR "green and blue infrastructure*" OR "blue infrastructure" OR aqueduct OR aqueducts OR wetland OR wetlands OR "urban waterway*" OR "riparian corridor*" OR lake OR lakes OR river OR rivers) AND ("physical activity" OR "physical activities" OR exercise OR exercises OR stress OR stresses OR "physical pressure" OR "physical pressures" OR "mental pressure" OR "mental pressures" OR anxiety OR anxieties OR worry OR worries OR nervousness OR apprehension OR " wellbeing" OR "well-being" OR psychological OR sleep OR sleeps OR bedtime OR "air pollutant" OR "air pollutants" OR "air pollution" OR "air quality" OR "particle pollution" OR "particulate matter" OR "social interaction*" OR "social behaviour" OR "social behaviours" OR "social relation*" OR "sense of community" OR "social cohesion" OR "social skills" OR "social skill" OR bonding OR "group cohesiveness" OR unity OR "social wellbeing" OR "social well-being" OR "socio-economic conditions" OR "socio-economic status" OR noise OR noises OR "sound pollution" OR "sound pollutant*")))
Cochrane Library	((green AND "blue space") OR "blue space*" OR canal OR "water body" OR "water bodies" OR (green AND "blue infrastructure") OR "blue infrastructure" OR (blue AND infrastructures) OR aqueduct OR aqueducts OR wetland OR wetlands OR "urban waterway" OR (riparian AND waterways) OR "riparian corridor" OR "riparian corridors" OR lake OR lakes OR river OR rivers) AND ("physical activity" OR "physical activities" OR exercise OR exercises OR stress OR stresses OR "physical pressure" OR "physical pressures" OR "mental pressure" OR "mental pressures" OR anxiety OR anxieties OR panic OR panics OR worry OR worries OR nervousness OR apprehension OR " wellbeing" OR " well-being" OR psychological OR sleep OR sleeps OR bedtime OR "air pollutant" OR "air pollutants" OR "air pollution" OR "air quality*" OR "particle pollution*" OR "particulate matter" OR "social interaction*" OR "social behaviour*" OR "social relation*" OR "sense of community" OR "social cohesion*" OR "social

	skill*" OR bonding OR "group cohesiveness*" OR unity OR "social wellbeing" OR "social well-being" OR "socioeconomic condition" OR "socioeconomic conditions" OR "socioeconomic status" OR "noise pollution" OR noise OR noises OR "sound pollution" OR (sound AND pollutant*)) in Title Abstract Keyword
EBSCOHOST/CINAHL	((TI "green and blue space" OR AB "green and blue space") OR TI "blue space*" OR AB "blue space*" OR TI canal OR AB canal OR TI "water body" OR AB "water body" OR TI "water bodies" OR AB "water bodies" OR (TI " green and blue infrastructure" OR AB "green and blue infrastructure") OR TI "blue infrastructure" OR AB "blue infrastructure" OR (TI blue infrastructures OR AB blue infrastructures) OR TI aqueduct OR AB aqueduct OR TI aqueducts OR AB aqueducts OR TI wetland OR AB wetland OR TI wetlands OR AB wetlands OR TI "urban waterway" OR AB "urban waterway" OR (TI riparian waterways OR AB riparian waterways) OR TI "riparian corridor" OR AB "riparian corridor" OR TI "riparian corridors" OR AB "riparian corridors" OR TI lake OR AB lake OR TI lakes OR AB lakes OR TI river OR AB river OR TI rivers OR AB rivers) AND (TI "physical activity" OR AB "physical activity" OR TI "physical activities" OR AB "physical activities" OR TI exercise OR AB exercise OR TI exercises OR AB exercises OR TI stress OR AB stress OR TI stresses OR AB stresses OR TI "physical pressure" OR AB "physical pressure" OR TI "physical pressures" OR AB "physical pressures" OR TI "mental pressure" OR AB "mental pressure" OR TI "mental pressures" OR AB "mental pressures" OR TI anxiety OR AB anxiety OR TI anxieties OR AB anxieties OR TI panic OR AB panic OR TI panics OR AB panics OR TI worry OR AB worry OR TI worries OR AB worries OR TI nervousness OR AB nervousness OR TI apprehension OR AB apprehension OR TI "wellbeing" OR AB "wellbeing" OR TI "well-being" OR AB "well-being" OR TI psychological OR AB psychological OR TI sleep OR AB sleep OR TI sleeps OR AB sleeps OR TI bedtime OR AB bedtime OR TI "air pollutant" OR AB "air pollutant" OR TI "air pollutants" OR AB "air pollutants" OR TI "air pollution" OR AB "air pollution" OR TI "air quality*" OR AB "air quality*" OR TI "particle pollution*" OR AB "particle pollution*" OR TI "particulate matter" OR AB "particulate matter" OR TI "social interaction*" OR AB "social interaction*" OR TI "social behaviour*" OR AB "social behaviour*" OR TI "social relation*" OR AB "social relation*" OR TI "sense of community" OR AB "sense of community" OR TI "social cohesion*" OR AB "social cohesion*" OR TI "social skill*" OR AB "social skill*" OR TI bonding OR AB bonding OR TI "group cohesiveness*" OR AB "group cohesiveness*" OR TI unity OR AB unity OR TI "social wellbeing" OR AB "social wellbeing" OR TI "social well-being" OR AB "social well-being" OR TI "socioeconomic condition" OR AB "socioeconomic condition" OR TI "socioeconomic conditions" OR AB "socioeconomic conditions" OR TI "socioeconomic status" OR AB "socioeconomic status" OR TI "noise pollution" OR AB "noise pollution" OR TI noise OR AB noise OR TI noises OR AB noises OR TI "sound pollution" OR AB "sound pollution" OR (TI sound pollutant* OR AB sound pollutant*))

Table S3: Inclusion / Exclusion Criteria

	**Inclusion Criteria:**	**Exclusion Criteria:**
**Population**	General human population	Non-human populations
**Intervention**	All inland waterways, coastal environments, canalled areas, blue infrastructure (BI), navigable transportation canals, aqueducts, lakes, marinas, rivers, ponds, reservoirs, marshes, estuaries, fountains, streams, reconstructed or recalibrated wetlands, waterfront parks, deculverted/daylighted areas, open air streams, urban waterways, riparian corridors, recalibrated urban parks, urban forests, natural preserves.	Studies looking at any other settings or virtual environments.
**Context**	All urban, peri-urban, rural environments	
**Outcome**	Studies with one or more of the outcomes measured being a direct effect on:<br>• Restoration<br>• Physical Activity<br>• Social Interactions<br>• Environmental Factors (temperature, noise, air pollution, etc.)<br>• Other health related causal pathways/mechanisms	• General studies about canals, green and blue infrastructure or green and blue spaces<br>• Studies without explicit mention of their direct impacts on health mediators (restoration, physical activity, environmental factors, social interaction).
**Type of Studies**	• Studies including scientific and empirical evidence.<br>• Quantitative studies with experimental or observational designs;<br>• Cross-sectional studies;<br>• Cohort studies;<br>• Longitudinal studies;<br>• Meta-analyses;<br>• Case studies of specific sites;<br>• Natural experiments;<br>• Prospective studies;	• Qualitative studies;<br>• Opinion pieces;<br>• Theoretical papers;<br>• Non-peer-reviewed studies

	• Randomised controlled trials;<br>• Case reports and series;<br>• Experiments conducted in controlled laboratory settings;<br>• Cross-over studies;<br>• Evaluation studies;	

Table S4: Quality Scores

Study	Quality Score (%)
Arbillaga-Etxarri et al., 2017 [31]	81.81%
Jansen et al., 2018 [32]	72.72%
Grow et al., 2008 [33]	77.27%
Jansen et al., 2017 [34]	95.45%
Karusisi et al., 2012 [77]	86.36%
Pasanen et al., 2019 [35]	95.45%
Perchoux et al., 2015 [78]	77.27%
Völker et al., 2018 [36]	86.36%
Wilson et al., 2011 [37]	100.00%
Ying et al., 2015 [38]	86.36%
Haeffner et al., 2017 [47]	90.90%
Zhou et al., 2017 [70]	90.90%
Arnberger et al., 2018 [71]	90.90%
Dzhambov, 2018 [79]	72.72%
Gascon et al., 2018 [39]	95.45%
Huynh et al., 2013 [40]	95.45%
Nutsford et al., 2016 [41]	90.90%
Pearson et al., 2019 [42]	90.90%
Rugel et al., 2019 [43]	95.45%
Triguero-Mas et al., 2017 [66]	86.36%
de Vries et al., 2016 [44]	90.90%
Triguero-Mas et al., 2015 [45]	81.81%
Reeves et al., 2019 [67]	80.76%
Benita et al., 2019 [72]	90.90%
de Bell et al., 2017 [46]	90.90%
Hipp et al., 2014 [73]	81.81%
Burkart et al., 2016 [48]	95.45%
Klok et al., 2019 [49]	54.54%
Kuehne et al., 2013 [50]	90.90%
Liu et al., 2018 [74]	72.72%
McNabola et al., 2008 [68]	67.85%
Miro et al., 2018 [51]	95.45%

Raso et al., 2009 [52]	86.36%
Saaroni and Ziv, 2003 [53]	58.33%
Smith and Moore, 2011 [54]	86.36%
Garrett et al., 2019 [60]	95.45%
Wu et al., 2019 [65]	86.36%
Lehnert et al., 2021 [64]	72.72%
Chen et al., 2021 [76]	81.81%
Amirbeiki and Ghasr, 2020 [63]	77.27%
Vert et al., 2020 [69]	95.45%
Subiza-Pérez, Vozmediano and San Juan, 2020 [62]	90.90%
Stieger, Aichinger and Swami, 2020 [75]	77.27%
Liu et al., 2020 [61]	86.36%
Wang, Ettema and Helbich, 2020 [59]	95.45%
Tan et al., 2021 [58]	81.81%
Hooyberg et al., 2020 [57]	90.90%
Garrett et al., 2020 [28]	95.45%
Chen and Yuan, 2020 [56]	86.36%
Roberts, van Lissa and Helbich, 2021 [55]	95.45%

Publisher's Note: MDPI stays neutral with regard to jurisdictional claims in published maps and institutional affiliations.
